# Hydro-Environmental Criteria for Introducing an Edible Halophyte from a Rainy Region to an Arid Zone: A Study Case of *Suaeda* spp. as a New Crop in NW México

**DOI:** 10.3390/plants10101996

**Published:** 2021-09-24

**Authors:** Francyelli Regina Costa-Becheleni, Enrique Troyo-Diéguez, Alejandra Nieto-Garibay, Luis Alejandro Bustamante-Salazar, Hugo Sergio García-Galindo, Bernardo Murillo-Amador

**Affiliations:** 1 Center for Biological Research of Northwest México SC (CIBNOR), Graduate Studies and Human Resources Program, Av. Instituto Politécnico Nacional No. 195, Colonia Playa Palo de Santa Rita Sur, La Paz 23096, Baja California Sur, Mexico; fbecheleni@pg.cibnor.mx; 2 Center for Biological Research of Northwest México SC (CIBNOR), Arid Zone Agriculture Program, Av. Instituto Politécnico Nacional No. 195, Colonia Playa Palo de Santa Rita Sur, La Paz 23096, Baja California Sur, Mexico; anieto04@cibnor.mx; 3 Department of Instrumental Analysis, Faculty of Pharmacy, University of Concepción (UdeC), Av. Víctor Lamas No. 1290, Concepción 4070386, Región del Bío Bío, Chile; lbustamante@udec.cl; 4 National Technological Institute of México (TecNM–Campus Veracruz), Av. Miguel A. de Quevedo No. 2779, Colonia Formando Hogar 91897, Veracruz, Mexico; hugo.gg@veracruz.tecnm.mx

**Keywords:** salt-marsh ecosystem, coastal vegetation, salt tolerance, euhalophyte, romerito, saline soils, salt marshes

## Abstract

Halophytes are capable of growing in saline environments. However, this attribute results from a wide genetic variability, making it difficult to approximate halophytes’ agroecological management. We examined the hydro-climatological attributes associated with the distribution of species of the genus *Suaeda* in NW Mexico and SW USA, and for *S. edulis* in central México. The analysis focused on the introduction of the semi-domesticated species *Suaeda edulis* as a new crop, from central regions of México, reaching an average yield of 8 Mg ha^−1^ of biomass, to arid NW México. The list of *Suaeda* species was elaborated from the eHALOPH and Calflora databases, and the NW México Herbarium Network. According to the Hydro-Environmental Availability Index (HEAI), the central regions of Mexico reflect a greater water availability, suitable for *S. edulis*. In such a humid region, HEAI varied from 6 to 18, indicating sufficient moisture for crops. In contrast, other *Suaeda* species, including *S. nigra*, *S. esteroa*, and *S. californica*, spread in NW Mexico and SW United States, where the water availability is null during the year, with HEAI scoring from 0 to 4. Under such dryness, *S. edulis* in NW Mexico will require water through optimized irrigation and plant breeding strategies to ensure its viability as a new crop.

## 1. Introduction

### 1.1. The Need to Introduce New Crops to Areas with Limited Water Availability

Introducing new crops with high water-use efficiency in drylands will help to curb the rising demand for water. Over a period of millions of years, through natural selection, only the most adapted species survived the desert environment. Xerophyte species have evolved, becoming well-adapted to extremely xeric conditions, developing physiological and morphological strategies of exploiting harsh environments that would desiccate other species [1]. Furthermore, halophytes and halotolerant crops are the only options for cultivation in saline-degraded soils. Thus, the use of halophytes may be a viable commercial alternative to ease pressure on the requirement for good quality land and water for conventional cropping systems, and for the utilization of land degraded by salinity [2].

### 1.2. Halophytes for Salty Soils Restoration and Saline-Water Based Agriculture

The term ‘halophyte’ (halos = salt; phyta = that has an affinity to) is ancient and has been used for more than 200 years to specify the plant species that developed in saline ecosystems, which are their natural habitat [3,4]. However, their wide environmental diversity and their strategies to cope with extreme adverse conditions or withstand excess salinity in the soil have resulted in variations in their definition [5,6,7]. Although these assumptions generate imprecision at a local and global level when classifying species as a halophyte [8], in recent decades, researchers reached an agreement when using different parameters, mainly sodium chloride (NaCl) concentrations, to identify species as halophytes [4]. Thus, the halophyte species have been described with higher precision at a global level [9,10,11].

Environmental impacts on ecosystems, such as the degradation and salinization of soils, motivate special attention being paid to halophytes; in previous studies, they were considered for various purposes, including the emerging “Biosaline-agriculture” [12,13,14], for restoring degraded or mining waste-contaminated soils [15,16]; for the bioremediation of soils and marginal waters necessary for the establishment and maintenance of green areas in arid, coastal, and desert areas [17]; as forage for raising livestock [18,19]; and as a biofilter for the removal of salts and residual contaminants from water [20,21]. Despite the relevance of this group of plants, it is a challenge at local and regional levels to identify and classify them, based mainly on their fundamental definition as plants adapted to living in saline-sodium soils and on their different uses [22].

### 1.3. The Genus Suaeda—Subfamily Chenopodiaceae—Family Amaranthaceae in México

In México, halophytes of the family Amaranthaceae and subfamily Chenopodiaceae are established in closed basins or bottoms of old salty lakes, which are frequently located on the coast, in coastal lagoons, in salt marshes, and on beaches. In arid and semiarid coasts, they survive and grow due to the contribution of salts they receive from the sea spray [23]. Changes in the content of sodium-salts in the soil horizons determine the variation of plant species established in the soil. These changes are due to the fluctuations in humidity and rates of soil desiccation, among other causes [24]. A particular case is the herbaceous *Suaeda edulis* (commonly named ‘Romerito’), an ancestral species domesticated in the central region of Mexico (Mexico City, Valley of Mexico) and adapted to saline ecosystems, is particularly noteworthy. ‘Romerito’ is important due to its commercial value when consumed as a fresh vegetable [25], which has aroused the interest of producers in northern and northwestern Mexico, because they face a significant problem of freshwater scarcity and gradual salinization of agricultural soils.

Because there are no antecedents for ‘Romerito’ in northwestern Mexico, previous studies on species other than *Suaeda* and other halophytes in this arid region are useful as a reference. Previous studies have been carried out by means of experimental observations and descriptions of native species associated with saline ecosystems [26,27,28], and assessments of physiological and morphological characteristics they possess for tolerating high degrees of salinity [29,30]. Current research regarding the diversity of the genus *Suaeda* and its species is focused on their distribution at the local and regional level.

### 1.4. Climate-Related Hydroenvironmetal Descriptors

Climate plays a central role in the distribution of plant species in salt-marsh ecosystems; primarily, woody and herbaceous species are characterized by different adaptations to water constraints [31]. For example, woody plant growth has been shown to be more sensitive to spatial variation in rainfall than that of herbaceous plants [32]. However, in rainy areas, other environmental factors gain importance in determining species distribution, whereas in arid zones, the growth of herbaceous species is frequently correlated with short-term moisture and energy-related variables [33], and requires lower precipitation. For the analysis of the distribution and adaptation of species to extreme conditions, temperature and rainfall data constitute a powerful tool because this numerical information is globally available [34].

Based on the above scenario and perspectives, the aim of this study was to examine a list of the halophytic species of the genus *Suaeda* dispersed in Northwest Mexico and Southwest United States from the available records of eHALOPH species [35], the Calflora web site [36], the Herbarium Network of Northwest Mexico (RHNM) [37], and the plants.usda.gov web site [38]. We aimed to propose the main hydro-environmental criteria for introducing their related species *S. edulis* from a rainy region in the central region of México to an arid zone of NW México, in order to diagnose the basic hydro-environmental requirements for its successful establishment.

The hypothetical premise of this research relates to the agroecological adaptation of *Suaeda edulis* as a consequence of its semi-domestication level, through its attributes that allow its cultivation in rainy zones, with biomass production of commercial value. These agroecological advances contrast with other *Suaeda* species well adapted to arid and semiarid zones because of their tolerance to salinity and drought. Accordingly, such *Suaeda* species adapted to northern latitudes due to their physiological strategies of adaptation to dry zones and saline soils, but produce lower biomass than *S. edulis* because of the lack of a domestication process. From the agroclimatological attributes of the production regions, a remaining question regarding the possible management of *S. edulis* is how to avoid possible water stress, which these species do not face in the central regions of Mexico due to their pluviometric regime.

## 2. Results

### 2.1. Suaeda Species Distribution in SW USA and NW Mexico

According to the plant descriptions in available databases, it was confirmed that *Suaeda* is a halophytic genus and can survive in high salted-marsh areas [35,36,37,38].

Sixteen *Suaeda* species (family Amaranthaceae, subfamily Chenopodiaceae) were reported to grow in coastal habitats and salt marshes of NW Mexico and SW United States; most of these are xerophytes and herbaceous plants with woody shrubs and above-ground shoots (Table 1). According to eHALOPH [35], Amaranthaceae is among the families with the highest number of halophytes, with 70 genera and 341 species. The genus *Suaeda* is ranked second among the family’s genera in terms of the number of species, and includes a total of 46 distributed species, corresponding to 3.48%. The listed species are distributed in six states in Northwest Mexico (Baja California, Baja California Sur, Chihuahua, Durango, Sinaloa, and Sonora) and in the southwestern states of the USA (Arizona, Nevada, Utah, and California) [32,36,37].

### 2.2. Climate and Soil Sustaining Halophytes in Northwestern and Central Mexico

According to the integration of the available information and inferring that *Suaeda* spp. are component species of the halophytic vegetation, their settlement zones in NW Mexico are characterized by their sandy or sandy-loam soils, with marine influence, depending on the flood contribution provided by cyclical tides, which are an important component of coastal salt marshes (Table 2). Because the humidity from precipitation in NW Mexico is very low and irregular, the coastal halophytes take advantage of the humidity from the tidal cycles, the ambient dew, and the sea breeze. In this context, the dominant vegetation in NW Mexico is sarcocaule, thorny scrub, and some annual grasses with reduced population densities; along coastal areas, vast areas of halophytes settle in floodplains [32,37].

Estuarine wetlands are a widespread group, but the hypersaline wetlands are more common along the north coasts of México. The euhaline systems are established in specialized localities scattered along both coasts. It is important to emphasize that human activities continue to threaten the coastal wetlands in Mexico. We verified in databases and official sources that halophytic species of the genus *Suaeda* of Northwest Mexico grow predominantly in habitats of flooded coasts with pioneer and ephemeral vegetation [40], coastal marshes and saline reed beds [41], salty-inland marshes [42,43], and on the Pacific coast [44,45]. Within the different types of species, other hydro-halophytes, xerophytes, and xerohalophytes are also described, with highly diversified life forms in relation to the climate and soil in which they are found, i.e., annuals, shrubs, and woody plants whose buds rest on or near the ground [46,47].

In the former Texcoco Lake area, the soils exhibit a saline-sodic condition due to factors and processes that favor salinization, which are the result of the interaction of natural events during the geological evolution of the Valley of Mexico basin. The current saline-sodic state of the soils is the result of the management of the lake since pre-Hispanic times, including the hydraulic works built for flood control, in addition to the effects of the growth of urban areas [48].

**Figure 1:**
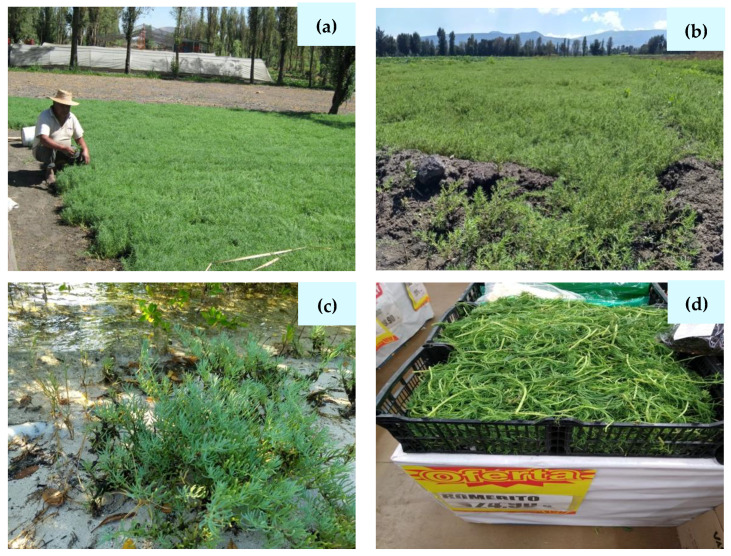
Photographs of *Suaeda* spp. (**a**) *Suaeda edulis* in an agricultural plot supervised by the owner; (**b**) panoramic view of a *Suaeda edulis* plot in the State of Mexico; (**c**) *Suaeda esteroa* on the coast of the Sea of Cortes in Baja California Sur, NW México; (**d**) *Suaeda edulis* ‘Romerito’ in a food market in the State of Sonora, Mexico. Photographs (**a**,**b**) were given and authorized by Dr. Roberto Noguez Hernández (Chapingo Autonomous University), Chapingo, Edo. Méx, México.

Figure 1 shows individuals, field plots, and specimens of *Suaeda* spp. from localities in central and northwestern regions of Mexico.

According to the evidence found in the records consulted, the climates and soils that support the ecosystems of *Suaeda* spp. differ between the central and northwestern regions of Mexico. Therefore, it appears likely that *S. edulis* may be successfully adapted in Northwestern Mexico, considering the pertinent adaptations with soil management and the definition of the planting periods.

### 2.3. Hydroenvironmetal Descriptors

**Figure 2:**
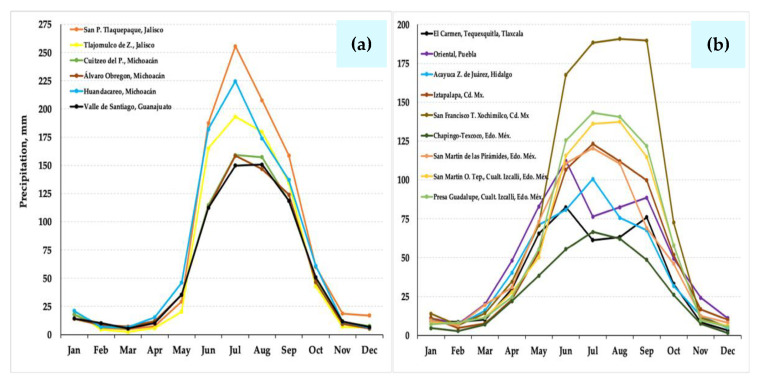
(**a**) Historical precipitation data from six stations in the western region of Mexico (Jalisco, Michoacán, and Guanajuato); and (**b**) from nine stations in the Valley of Mexico and the central-eastern region of Mexico (Tlaxcala, Puebla, Hidalgo, Mexico City and Mexico State), where *Suaeda edulis* is reported.

**Figure 3:**
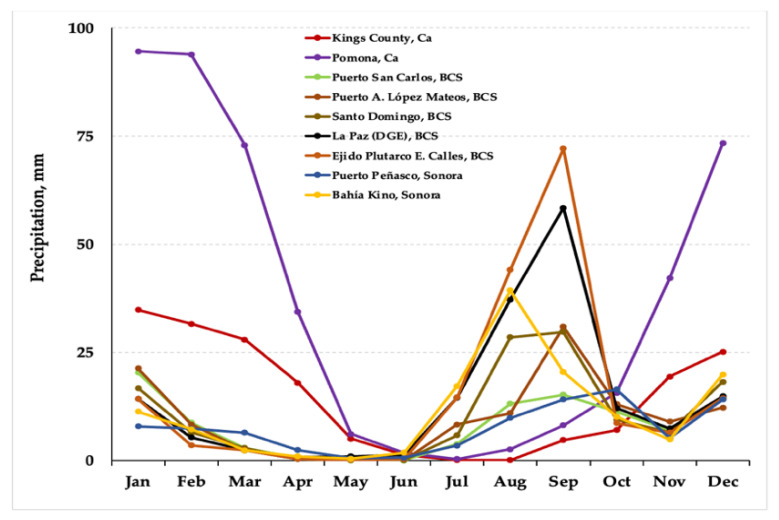
Historical precipitation data from nine stations in NW Mexico (Baja California Sur and Sonora) and the SW region of the USA (California), where several species of *Suaeda* are reported.

Historical precipitation (pp) data were analyzed for six localities in western México, including the states of Jalisco (San Pedro Tlaquepaque and Tlajomulco de Zuñiga), Michoacán (Cuitzeo del Porvenir, Álvaro Obregón, and Huandacareo), and Guanajuato (Valle de Santiago); and at nine stations in the Valley of Mexico and central-eastern Mexico: one in Tlaxcala State (El Carmen Tequexquitla), one in Puebla State (Oriental), one in Hidalgo State (Acayuca, Zapotlán de Juárez), two in Mexico City (Iztapalapa and San Francisco Tlalnepantla, Xochimilco), and four in the State of Mexico (Chapingo, Texcoco; San Martín de Pirámides; San Martín Obispo Tepetixpan, Cuautitlán Izcalli, and Presa Guadalupe, Cuautitlán Izcalli) [49]. It is noticeable that *S. edulis* ecotypes settle in localities of Western México with a rainy regime, where pp is from 200 to 250 mm month^−1^ during the humid season, and from 120 to 190 mm in the Valley of México and Central States (Figure 2). In contrast, localities in NW México and SW United States [50], where other species of *Suaeda* other than *S. edulis* have develop, register a notably low pp, oscillating from 0 to 90 mm month^−1^ during most of the year (Figure 3) [31]. These species include *S. californica*, *S. nigra*, *S. esteroa*, *S. maritima*, and *S. fruticosa* [35,36]. Because of the dry prevailing climate [51], the dominant vegetation in NW Mexico is typical of arid zones, and along the coast, halophytes settle in floodplains [52,53].

**Figure 4:**
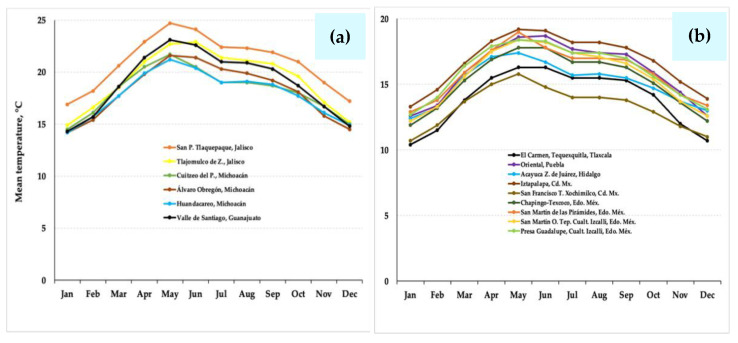
(**a**) Historical mean temperature data from six stations in the western region of Mexico (Jalisco, Michoacán, and Guanajuato); and (**b**) from nine stations in the Valley of Mexico and the central-eastern region of Mexico (Tlaxcala, Puebla, Hidalgo, Mexico City, and Mexico State), where *Suaeda* edulis is reported.

**Figure 5:**
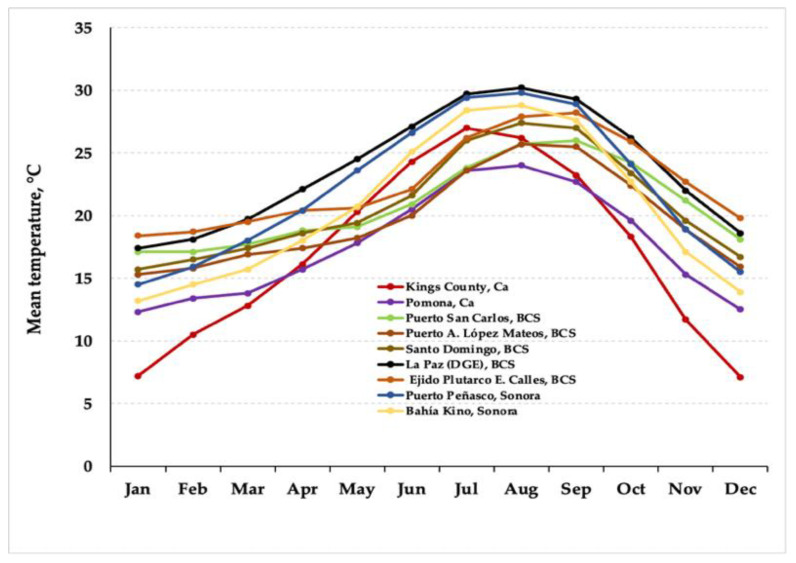
Historical mean temperature data from nine stations in NW Mexico (Baja California Sur and Sonora) and the SW region of the USA (California), where several species of *Suaeda* are reported.

Temperature data were also analyzed for the above-mentioned localities: six stations in western Mexico (Jalisco, Michoacán, and Guanajuato), nine stations in the Valley of Mexico and the Central States of México (Estado de México, CDMX, Hidalgo, Puebla, and Tlaxcala), oscillating from 12 to 24.5 °C [49] (Figure 4). Here, the temperature is relatively low with a temperate regime. This causes less thermal stress than the environmental conditions prevailing in NW México, where the monthly mean temperature fluctuates from 7 to 30 °C, reflecting aridity with extreme thermal conditions and higher temperature variations [50,54,55] (Figure 5), and thus motivating a greater thermal adaptation of *Suaeda* species.

**Figure 6:**
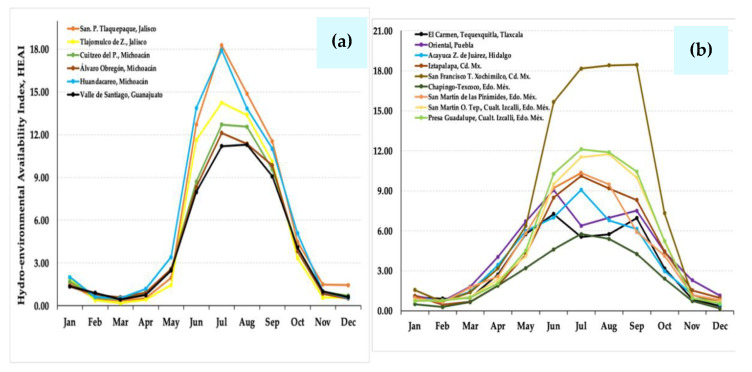
(**a**) Historical data of the Hydro-Environmental Availability Index (HEAI) from six stations in the western region of Mexico (Jalisco, Michoacán, and Guanajuato); and (**b**) from nine stations in the Valley of Mexico and the central-eastern region of Mexico (Tlaxcala, Puebla, Hidalgo, Mexico City and Mexico State), where *Suaeda edulis* is reported.

**Figure 7:**
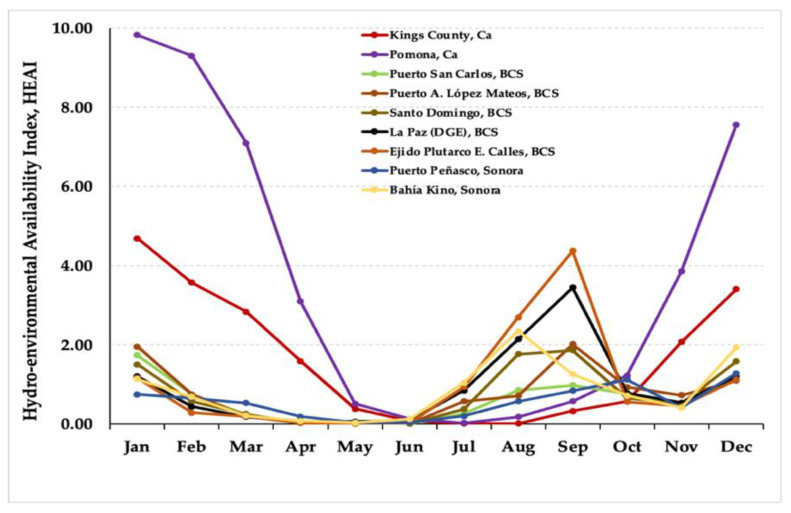
Historical data of the Hydro-Environmental Availability Index (HEAI) from nine stations in NW Mexico (Baja California Sur and Sonora) and the SW region of the USA (California), where several species of *Suaeda* are reported.

According to the precipitation gradients and temperature oscillations in the settlement localities, the climate of the localities where *Suaeda* has developed varies from the dry subtropical to the extremely arid type, including the sub-dry Mediterranean type, whose gradients are expressed and delimited by the De Martonne Index [56]. This index has been numerically modified to calculate an innovative Hydro-Environmental Availability Index (HEAI), as suggested for arid zone studies [57] (Figure 6; Figure 7).

In relation to the Hydro-Environmental Availability Index (HEAI), a greater water availability is reflected in the western regions of Mexico, the Valley of Mexico, and central-eastern states, which is emphasized in the rural areas of Mexico City. Accordingly, such areas exhibit a favorable climate for the cultivation of *S. edulis* (‘Romerito’), which grows with optimal conditions under rainfed management or irrigation management. In the aforementioned humid region, we observed that the HEAI reached scores from 6.0 to 18.0 for about six months, indicating a suitable climate for the cultivation of the plant (Figure 6). In contrast to the reported distribution of *S. edulis* in these regions, other *Suaeda* species, including *S. californica*, *S. nigra*, *S. esteroa*, *S. maritima*, and *S. fruticosa*, are found in northwestern Mexico throughout the states of Baja California Sur and Sonora, and in the southwestern United States in the state of California. In these areas, very low water availability—practically null during most of the year—is observed, which is a favorable climate for the growth of *Suaeda* species adapted to dry regions. It is observed that the HEAI, reached scores from 0 to 4 (Figure 7), characterized by the predominance of dry-semidry climates, including desert.

**Figure 8:**
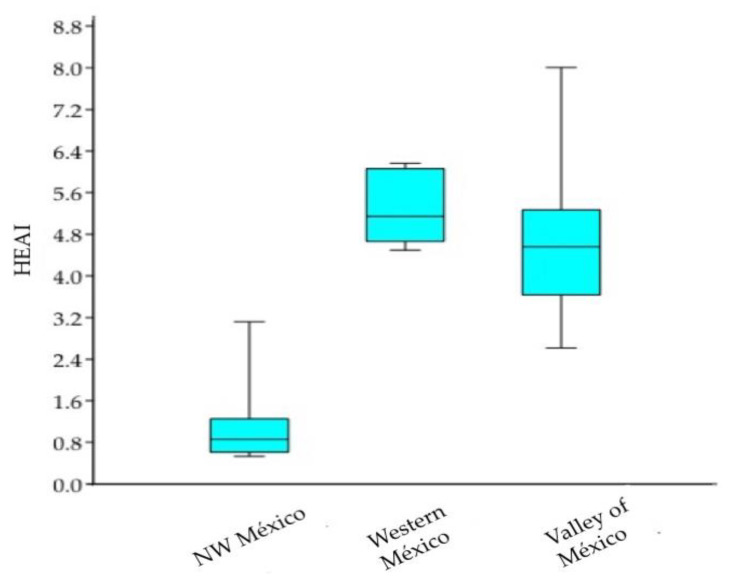
ANOVA graphical display for HEAI grouped in three regions of *Suaeda* spp. settlement.

The ANOVA performed to compare the three regions of establishment of *Suaeda* spp. considering 24 localities, corroborates highly significant differences between the groups analyzed, proving with a high level of significance the low water availability prevailing in northwestern Mexico (F: 34.23, *p* < 0.01) (Figure 8).

The obtained results suggest that, to successfully introduce and settle *S. edulis* as a new crop in NW México, it will be necessary to design hi-tech irrigation systems, such as drip, hydroponics, and protected agriculture, to achieve appropriate moisture conditions and the microclimate for the cultivation of the vegetable.

## 3. Discussion

Sixteen *Suaeda* species of the family Amaranthaceae grow in coastal habitats and salt marshes, distributed in regions of northwestern Mexico and southwestern United States, as reported in the eHALOPH and Calflora databases, and the Network of Herbariums of Northwest Mexico. Most of these are reported as xerophytes and/or halophytes, and herbaceous plants with woody shrubs and above-ground shoots, grown in coastal habitats and salt marshes, distributed in the semiarid regions of northwestern Mexico and southwestern United States.

According to the HEAI, the western region and the Valley of Mexico reflect a greater water availability, where Mexico City exhibits a favorable climate in which *Suaeda* (‘Romerito’) crops may be grown and developed with optimal conditions. On the contrary, with the prevailing dry-soil conditions, the eventual implementation of *S. edulis* cultivation in NW Mexico requires the design of adequate irrigation systems and sustainable water management. In addition, plant breeding strategies are necessary to ensure its viability as a new crop in these arid areas [58].

In relation to the eventual water demand required for the cultivation of *Suaeda edulis* following its introduction to the arid zone of northwestern Mexico, it is important to consider the evapotranspirative demand of the microclimate in its place of origin (Valley of Mexico, central and eastern states). For example, for the locality of Chapingo, Texcoco, a total annual evaporation of 1647.6 mm is reported, with monthly evaporations of 125.1, 117.3, 121.5, 117.5 and 108.9 mm, for the months of August to December, respectively. These data indicate an average daily evaporation for the year of 4.5 mm. In turn, for the growing period from August to December, the daily water demand is estimated to be 3.86 mm (Conagua), which should be met by means of an appropriate irrigation system.

Regarding the possible impacts related to the change in land use, the identification of this eventual local modification must be undertaken through photointerpretation with field verification, whose results must be processed in a geographic information system. Accordingly, to consider *S. edulis* as an emergent or optional resource, a careful inventory of the vegetation and land use must be carried out to determine the composition and distribution of the areas susceptible to exploitation [59]. Environmental impacts on natural resources must also be anticipated; possible modifications or alterations to the natural resources and their environment as a result of the introduction of *S. edulis* are presented in Table 3; Table 4.

For *Suaeda edulis* (‘Romerito’), the Ministry of Agriculture and Rural Development of México (SADER) reported an agricultural production of 129.0 Mg was achieved in a planted area of 17 ha with an average yield of 8.0 Mg ha^−1^ of edible biomass, during the spring-summer 2020 agricultural cycle in México City. Other municipalities within the region and rural localities surrounding México City also reported cropping areas totaling about 750 ha (Ministry of Agriculture and Rural Development of México ‘SADER’ [60].

To establish useful guidelines for agroecological planning in the region where the new crop will be introduced, and also to prevent possible variations due to climate change [61], it is necessary to establish parameters and criteria to help identify and address agri-environmental compatibility problems and limitations. In the present study, and in view of the lack of previous experience with *Suaeda edulis* in northwestern Mexico, we propose the list of criteria shown in Table 3, where we emphasize that periods of high temperatures will limit crop cycles to periods that reflect less thermal stress.

By integrating the numerical information generated and the data consulted, a list of environmental links and possible implications derived from the introduction of *Suaeda edulis* in the northwestern region of Mexico is presented (Table 4). Among the most important aspects to consider is the compatibility of the species with the availability of saline soils, in addition to its value as a crop that, in principle, will not require fresh water or compete with conventional crops for the availability of water resources.

## 4. Materials and Methods

The distribution of the bushy-halophyte *Suaeda* spp. in NW Mexico and SW United States of America was analyzed, with site verifications in the southern portion of the Baja California peninsula, Mexico, from October 2019 to September 2020. Reference localities were determined for the states of Baja California, Baja California Sur, and Sonora in NW Mexico, and in the state of California in SW United States of America, for their analysis, given the locations’ thermo-pluviometry regime that characterizes their zones of settlement.

### 4.1. Study Regions

Northwest México - Areas of dispersion and establishment of *Suaeda* spp. In Northwest Mexico, plant resources and ecosystems are conditioned by the environmental traits, including hot weather and dry soils, imposed by the Sonoran Desert, located between 23 to 35° LN and 109 to 117° LW. This area covers 260,000 km^2^, in the southern half of Arizona and southwest California in the United States, most of the Baja California Peninsula, some islands in the Gulf of California, and a large proportion of the state of Sonora [31]. Compared with the three other deserts existing in North America (the Chihuahuan, the Great Basin, and the Mojave), the Sonoran Desert is the northwesternmost Mexican territory, the wettest and warmest of the four, and possibly the most diverse from the botanical perspective [32]. The dominant climate in northwestern México is semiarid; the historical rainfall ranges from 100 to 320 mm per year, with an average annual temperature oscillating from 24 to 33 °C. Agriculture in this region is important, not only positively due to its economic contribution, but also negatively as evidenced by environmental deterioration. The main effect is expressed on the water depletion caused by overexploitation of aquifers and soil salinity [58].

**Figure 9:**
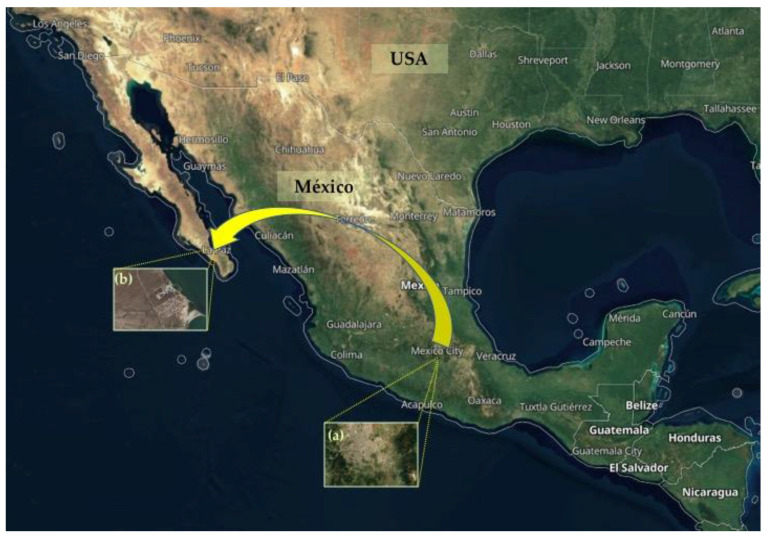
Geographical location of the region of: (**a**) Valley of Mexico, México City, and central Mexican States, where the semi-domesticated halophyte *S*. *edulis* (known as ‘Romerito’) is cultivated; (**b**) La Paz BCS, the target locality in NW México for the introduction of *S. edulis*.

Central México - Areas of settlement of *Suaeda edulis.* The semi-domesticated halophyte *Suaeda edulis* (‘Romerito’), the closest related reference to *Suaeda* wild species, is cultivated in central Mexico, in rural areas of Mexico City, and the surrounding areas of Lake Texcoco. These areas used to be a lake zone where, prior to pre-Hispanic times, there was a lagoon complex consisting of five interconnected bodies of water. The largest of these was the lake of Texcoco and, to the south, the lakes of Xochimilco and Chalco, covering portions of Mexico City and the State of Mexico. *S. edulis* is cultivated in saline soils of the Valley of Mexico, central States, and western Mexico [25,40] (Figure 9).

### 4.2. Geography, Climate and Soils of Northwest México

The central portion of the Baja California Peninsula is characterized by the Vizcaino Desert, covering an extensive area between two seas, the Gulf of California, and the Pacific Ocean, with abundant sand dunes, halophyte vegetation, coastal lagoons, and floodplains [43]. In the Mexican mainland, the Altar Desert locates in the northwestern portion of Sonora, which also covers the Pinacate region, one of the driest regions in the world, where annual precipitation ranges from 50 to 100 mm. The annual pp in the Sonoran Desert varies between 100 and 300 mm; it is distributed irregularly throughout the year, gradually increasing towards the south. Rains are registered mainly in summer, from July to September, when maximum temperatures exceed 40 °C in the shade, and the climate is controlled by the North American Monsoon [44,45]. Vegetation is an integrator of climate, soil, geomorphology, and environmental history, and its dominant physiognomy, together with data on the geographic distribution of indicator species, have been used as the fundamental criterion for recognition of regional borders [46].

### 4.3. Distribution of the Genus Suaeda Species in Northwest Mexico

In this study, the eHALOPH and Calflora databases, and the Northwest Mexico Herbarium Network (RHNM) and the USDA plants databases, were used as reference sources for the halophyte species of the genus *Suaeda* in northwest Mexico and southwest United States of America [35,36,37,38]. The information that is registered in eHALOPH is based on a total relation of halophyte plant species according to an update of data through studies carried out by different experts [47,48]. The concept described for the halophyte species included in this database is related to the ability to tolerate high values of electrical conductivity (EC), from 4 dS m^−1^ during the relevant stages of their growth, which is at the moment the unit is used to measure the salinity of the saturated extract (NaCl) [47]. The RHNM is integrated with the collaboration of different universities and their respective herbaria, including the Autonomous University of Baja California (BCMEX), Center for Biological Research of the Northwest (HCIB), University of Sonora (USON), Autonomous University of Sinaloa (UAS), Food and Development Research Center–Mazatlán Unit (HCIAD), and the Interdisciplinary Research Center for Integral Regional Development Durango Unit (CIIDIR-Durango), available on-line [37,49].

### 4.4. Thermo-Hydroenvironmetal Descriptors

For the analysis of distribution and adaptation of *Suaeda* species to extreme conditions, temperature and rainfall data were applied for estimating the hydro-environmental indicators associated with their establishment and settlement. Climate data were obtained from the official database of the National Water Commission of Mexico [49], and from the WorldClimate on-line databases [50].

For local climate variability analysis, the Martonne Index (IM) was applied because it is widely used due to its simplicity, requiring only monthly rainfall and temperature data [56]; this index is expressed by Equation (1):

IM = (12 * pp)/(t + 10)(1)

where pp is the monthly precipitation (mm) and t is the average monthly temperature (°C); 12 is a constant for the application of the model to the analysis of monthly data.

When the monthly IM is greater than 20, it is considered a humid month; a value ranging from 10 to 20 corresponds to a semiarid month; and an arid month is when the index has a value less than 10. The difficulty in the application of the IM relates to the challenge in making comparisons of quantitatively similar stations or regions. In addition, it reflects a rainfall condition rather than aridity or drought, because its value increases in direct proportion to the magnitude of pp. To make an appropriate adjustment to the IM, this equation was modified to an alternative numerical model that was highly correlated with water deficit and was also sensitive to minimum precipitation values. This alternative indicator, modified from the IM equation, was defined by Equation (2):

HEAI = Ks (12 * pp)/(t + 10)(2)

where HEAI is the Hydro-Environmental Availability Index, pp is the monthly precipitation in mm, t is the average monthly temperature in °C and Ks is a dimensionless coefficient of scale adjustment, with a value of 0.193.

## 5. Conclusions

Water for agriculture in northwestern Mexico is being depleted. Thus, a significant reduction in agricultural areas is expected in the near future. Similarly, the soils in the Northwest are undergoing a gradual salinization process, which may affect 40% of the agricultural surface in the next decade. Therefore, it is necessary to increase the availability of crops tolerant to salinity and extreme environmental conditions (high temperatures, low rainfall). *Suaeda edulis* (‘Romerito’) is a salt-tolerant plant with commercial importance, established in central and western Mexico, where the climatic regime is dominated by moderate temperatures and high rainfall, facilitating rainfed cultivation. Although it shows potential suitability as a new crop, currently *S. edulis* is not grown in northwestern Mexico. At present, there is a lack of references for this species. Thus, based on the other species of the genus *Suaeda*, it is important to determine the parameters and agroclimatic thresholds of the adapted *Suaeda* species.

*S. edulis* ‘Romerito’ in Central and Western Mexico produces 8.0 Mg ha^−1^ of biomass, under humid conditions of monthly pp up to 255 mm, with at least five months above 100 mm, and a monthly temperature oscillating from 4 to 24.5 °C. *Suaeda* species other than *S. edulis* in Northwest Mexico and the Southwest of the United States receive monthly rainfall from 0 to 92 mm, with only two months above 90 mm, and a temperature from 7 to 30.5 °C. Precipitation in the Center and West is four times higher than that in Northwest Mexico. Thus, it is important to plan for water supply strategies, such as drip irrigation, protected agriculture, hydroponics, and aquaponics. Various challenges have to be addressed to create knowledge for planning the sustainable use of *Suaeda* species, as a natural resource with potential use in the agri-food industry of semiarid coastal areas.

It should be noted that halophytes may represent the last viable option to be cultivated in highly saline soils or under low quality water conditions. Several studies have previously demonstrated their potential uses and value for the agri-food sector [62].

## Figures and Tables

**Table 1:** Distribution of 16 halophytic species of the genus *Suaeda*, family Amaranthaceae (species are listed in alphabetical order).

Species	Distribution
*Suaeda calceoliformis*	California (USA), Great Salt Lake, Utah (USA)
*Suaeda californica*	Pacific coast (MEX), Southwest (USA)
*Suaeda conferta*	Pacific coast (MEX)
*Suaeda esteroa*	West coast (USA), Balandra BCS (MEX)
*Suaeda fruticosa*	Pacific coast, BCS (MEX), Bahía Kino, Sonora (MEX)
*Suaeda jacoensis*	Northwest (MEX), Southwest (USA)
*Suaeda maritima*	Pacific coast (MEX/USA), Atlantic coast (USA)
*Suaeda mexicana*	Northwest (MEX), Southwest (USA)
*Suaeda moquinii*	California coast (USA)
*Suaeda nigra*	West coast (USA), Colorado Desert (USA), NW (MEX), SW (USA), Mojave Desert (USA), Great Basin (USA), California (USA),
*Suaeda nigrescens*	Chihuahuan Desert (MEX), Southwest (USA)
*Suaeda prostrata*	West coast (USA), Pacific coast (USA/CAN)
*Suaeda puertopenascoa*	Northwest (MEX), Sea of Cortés coast (MEX)
*Suaeda suffrutescens*	NW (MEX), SW (USA), Mojave Desert (USA), Great Basin (USA)
*Suaeda taxifolia*	Northwest (MEX)
*Suaeda torreyana*	West coast (USA), Lower Colorado Desert (USA)

Sources: eHALOPH [35]; Calflora [36]; RHNM [37]; plants.usda.gov [38]; Khan et al. [39].

**Table 2:** Habitat and plant traits of 16 halophytic species from the genus *Suaeda* spp., Amaranthaceae family, widespread in NW México and SW United States.

Species	Habitat	Plant Type	Life Form	Source
*Suaeda calceoliformis* (Hook.) Moquin	Native coastal habitats; coastal strand; wetland	Hydro-halophyte	Annual herb	eHALOPH [35], calflora.org [36], RHNM [37], plants.usda.gov [38]
*Suaeda californica* S. Watson	Salt marsh; coastal areas; wetlands	Hydro-halophyte	Subshrubs, shrubs, woody plants	eHALOPH [35], calflora.org [36], plants.usda.gov [38]
*Suaeda conferta* (Small) I.M. Johnst	Subtropical and trop. coastal areas–NE México (Tamaulipas)	Xero-halophyte	Subshrubs, shrubs, woody plants	eHALOPH [35]
*Suaeda esteroa* Ferren & S.A. Whitmore	Coastal salt marsh and saline streams; sub-humid wetlands	Hydro-halophyte	Shrub, perennial herb	calflora.org [36]
*Suaeda fruticosa* Forssk. ex J.F. Gmel	Salt marsh; coastal areas	Xero-halophyte	Chaemaephyte	eHALOPH [35], RHNM [37]
*Suaeda jacoensis* I.M. Johnst	Periodically flooded shorelines with pioneer/ephimer vegetation	Xerophyte	Chaemaephyte, shrub, subshrub, woody plants	eHALOPH [35]
*Suaeda maritima subsp. maritima* (L.) Dumort	Coastal salt marsh; coastal wetlands	Hydro-halophyte	Shrub, perennial herb	RHNM [37]
Suaeda mexicana Standl	Subtropical, semiarid and arid lands	Xerophyte	Chaemaephyte, shrub, woody plant	eHALOPH [35]
*Suaeda moquinii* (Torr.) Greene	Saline lake	Hydro-halophyte	Herb, shrub, subshrub	eHALOPH [35], plants.usda.gov [38], Khan et al. [39]
*Suaeda nigra* (Raf.) J.F. Macbr	Coastal salt marsh	Xerophyte	Subshrub, shrub, woody plant	calflora.org/ [36]
*Suaeda nigrescens* I.M. Johnst	Estuaries; saline coastal lagoons	Xerophyte	Chaemaephyte	eHALOPH [35]
Suaeda prostrata Pall	Interior salt marshes	Xero-halophyte	Annual	eHALOPH [35]
*Suaeda puertopenascoa* M.C.Watson & W.R.Ferren	Coastal salt-marsh-Northwest Sonora (Puerto Penasco)	Xero-halophyte	Subshrub, shrub	RHNM [37]
*Suaeda suffrutescens* S. Watson	Estuaries; saline coastal lagoons	Xerophyte	Chaemaephyte, shrub	eHALOPH [35]
*Suaeda taxifolia* (Standl.)	Estuaries-southern California, NW México (Baja California)	Xero-halophyte	Subshrub, shrub	RHNM [37]
*Suaeda torreyana* S. Watson	Estuaries; saline coastal lagoons	Xerophyte	Shrub	eHALOPH [35]

**Table 3:** Hydro-environmental criteria and possible problems or limitations to be addressed as a consequence of introducing *Suaeda edulis* (‘Romerito’) from temperate rainy zones to a semiarid region of Northwest México.

Hydroenvironmental Criteria	Problem or Limitation in the Target Region	Strategy for Solution/Mitigation	Warning Status
1. Precipitation	Soil moisture is deficient for crops, and the sources of irrigation water are depleted.	Design efficient irrigation with the possibility of using saline or residual waters.	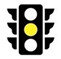
2. Temperature	High temperatures and solar radiation prevail in the region and agricultural localities.	A suitable season must be assessed for *S. edulis*, i.e., winter, spring.	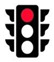
3. HEAI	High temperatures associated with low humidity magnify heat stress and water deficit.	Select the best growing seasons combined with water conservation strategies.	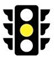
4. Water quality	No problems with water quality have been detected, since the crop is grown in saline soils.	Carefully manage poor quality water to avoid soil degradation.	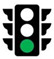

**Table 4:** Environmental links and possible implications associated with the introduction of *Suaeda edulis* (‘Romerito’) from temperate rainy zones to a semiarid region of Northwest México.

Environmental Issue	Origin: Temperate Rainy Regions.-Valley of México, México City, Western Regions.	Target: Semiarid Dry Zones.-Northwest México: Baja California Sur (La Paz BCS).	Agroecological Significance
1. Freshwater demand	Low demand, without impact on water resources; normal pp supplies enough moisture; irrigation only on demand.	Real possibilities of irrigation with saline water; it would not increase the demand for fresh water for a new crop.	Highly positive because it 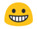 would not increase the freshwater demand for an introduced new crop.
2. Biodiversity	As traditional crop, ‘Romerito’ increases biodiversity within crops and agroecosystems; enthomofauna, birdlife, etc.	There are wild ecotypes of *Suaeda* with unknown potential, only used by cattle during drought periods.	It is positive, but it is 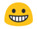 highly advisable to verify field trials for observation.
3. Land-use	It is a monoculture in small plots but is part of diversified regional agroecosystems.	Implications not yet known, but it should be planned to be irrigated with saline water.	It is positive if soils are 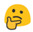 properly diagnosed, conserved and managed.
4. Soil or substrate quality requirements	It stabilizes saline soils with proper management; it is cultivated where other crops can not grow and produce..	Similar to its place of origin, it should be used in degraded soils unsuitable for other crops, as a feasible option.	Positive if not disturbing 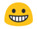 good quality lands for conventional crops; needs proper selections of soils.
5. Surrounding soil quality	It is grown mainly in saline soils; it does not affect surrounding soils.	Attention should be paid to discharge areas, so as not to salinize undisturbed soils.	Neutral but with a risk 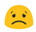 potential; soil quality needs to be monitored.
6. Harvest–marketing	Markets are specialized and important in specific seasons.	New local markets must be developed and promoted.	At this time it is negative; 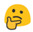 new markets are needed.
7. Harves–economic profitability	It is produced for decades, its profitability is reached in central states, México City and in the Valley of México.	The economics of producers owning salt-affected soils are in decline, so this may be an option to regain profitability.	At this time it is unknown; 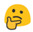 detailed analysis are needed, but it is expected to be an option for saline soils, actually useless.
8. Potential risk of becoming a weed	It has not become a weed, but it associates positively to other halophytes in untillaged soils.	No risk as a weed is detected, since it does not have asexual propagation structures.	If grown only on saline 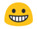 soils or in protected agriculture, no escape would be expected.

## Data Availability

Data of precipitation and temperature of the study sites can be found in the climatological web page of the National Water Commission of Mexico: https://smn.conagua.gob.mx/es/climatologia/informacion-climatologica/normales-climatologicas-por-estado, accessed on 25 March 2021.
